# Utilization of cardiac tests in anthracycline‐treated cancer survivors differs between young adults and children: A claims‐based analysis

**DOI:** 10.1002/cam4.6801

**Published:** 2023-12-09

**Authors:** Xu Ji, Xin Hu, Joseph Lipscomb, Eric J. Chow, Ann C. Mertens, Sharon M. Castellino

**Affiliations:** ^1^ Department of Pediatrics Emory University School of Medicine Atlanta Georgia USA; ^2^ Aflac Cancer & Blood Disorders Center, Children's Healthcare of Atlanta Atlanta Georgia USA; ^3^ Department of Public Health Sciences University of Virginia School of Medicine Charlottesville Virginia USA; ^4^ Department of Health Policy and Management, Rollins School of Public Health Emory University Atlanta Georgia USA; ^5^ University of Washington School of Medicine and Fred Hutchinson Cancer Research Center Seattle Washington USA

**Keywords:** anthracycline, cardiac testing, commercial claims data, survivors of childhood and adolescent cancer

## Abstract

**Background:**

The Children's Oncology Group Guidelines recommend a cardiacechocardiogram, or comparable functional imaging, following therapy completion in survivors of childhood/adolescent cancers exposed to anthracyclines.

**Methods:**

Using the 2009–2019 Merative™ MarketScan® Commercial Database, we examined real‐world utilization of cardiac testing among 1609 anthracycline‐treated survivors of childhood/adolescent cancers.

**Results:**

The cumulative incidence of receiving an initial cardiac test by 5.25 years from the index date (six months after end‐of‐therapy) was 62.3% (95% CI = 57.5%–66.7%), with median time to initial test being 2.7 years (95% CI = 2.5%–3.1%). Young adults (18–28 years) were less likely than children (≤17 years) to receive cardiac testing (hazard ratio [HR] = 0.42, 95% CI = 0.3%–0.49%). More likely to receive cardiac testing were survivors receiving hematopoietic stem cell transplantation versus chemotherapy only (HR = 2.23, 95% CI = 1.63%–3.03%), and survivors with bone or soft tissue versus hematologic cancer (HR = 1.64, 95% CI = 1.30%–2.07%).

**Conclusions:**

Nearly 40% of anthracycline‐treated survivors of childhood/adolescent cancers had not received cardiac testing within 5.25 years post‐index date, with young adults least likely to receive a test.

## Graphical abstract

In this nationwide, insurance claims data‐based study, we examined the real‐world utilization of cardiac tests after therapy completion among anthracycline‐treated survivors of childhood and adolescent cancer. We found that nearly 40% of anthracycline‐treated survivors at risk of cardiac dysfunction had not received any cardiac test within 5.25 years from 6 months after end‐of‐therapy, despite national guidelines detailing the importance of timely screening for early detection of cardiac dysfunction. Young adult survivors were least likely to receive cardiac testing despite having insurance.

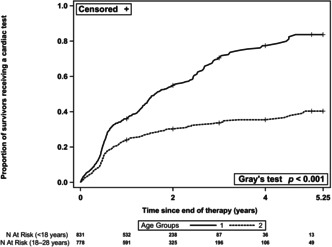

## 1 INTRODUCTION

Survivors of childhood and adolescent cancer exceed 500,000 in the United States, and this population continues to grow.^1^ Survivorship guidelines recommend screening for cardiotoxicity associated with therapeutic exposure in childhood and adolescent malignancies.^2^, ^3^, ^4^ Specifically, the Children's Oncology Group (COG) Guidelines recommend a cardiac echocardiogram, or comparable functional imaging, following therapy completion in survivors exposed to anthracyclines.^2^ There is currently a dearth of evidence on posttreatment uptake of guideline‐recommended cardiac testing in exposed survivors. Existing studies used self‐report data, focused on clinical trial settings, or else were not United States‐based.^5^, ^6^, ^7^ Using nationwide insurance claims data, we examined real‐world utilization of cardiac testing following therapy completion among anthracycline‐treated survivors of childhood and adolescent cancers.

## 2 METHODS

We used the 2009–2019 Merative™ MarketScan® Commercial Database, a nationwide convenience sample of outpatient, inpatient, and pharmacy insurance claims from United States employer‐sponsored health plans.^8^, ^9^ We identified survivors who (a) received anthracyclines for hematologic cancers or bone and soft tissue cancers (aged ≤21 years at cancer diagnosis; Table S1); (b) completed cancer therapies; and (c) remained continuously insured for ≥1 year from their index date, defined as 6 months after the end‐of‐therapy date (Figure S1). In a sensitivity analysis that modified the definition of index date to the end‐of‐therapy date, we found similar results (available upon request). End‐of‐therapy refers to 30 days following either the last date of inpatient or outpatient claims for cancer therapies or the date when the last oral chemotherapy prescription is filled, whichever is later.^10^, ^11^, ^12^ We followed the algorithm applied in previous claims data‐based studies to identify hematologic cancers and bone and soft tissue cancers,^10^, ^11^, ^12^ which represent the majority of anthracycline‐treated childhood and adolescent cancers.^13^ Specifically, cancer diagnoses were identified using the International Classification of Diseases (ICD) codes, and cancer therapies (surgery, chemotherapy, radiotherapy, and hematopoietic stem cell transplantation) were identified using procedure codes and the National Drug Codes.^10^, ^11^, ^12^

We used the Kaplan–Meier method to estimate the cumulative incidence of receipt of the first cardiac test—including echocardiogram, cardiac magnetic resonance imaging, and/or multiple gate acquisition scan (Table S2)—within 5.25 years post index date, allowing a leeway of 0.25 year (3 months) for potential delays in scheduling medical appointments as the 5‐year cut‐point for adherence approaches. Censoring occurred at the end of a year if survivors discontinued insurance enrollment in the following year, or at the end of our study period (December 31, 2019). Cumulative incidence curves were generated for all survivors, as well as among subgroups stratified by key sociodemographic characteristics that may contribute to differential utilization of cardiac tests.

Cox proportional hazards regressions were used to identify survivor characteristics that were significantly associated with the first cardiac test receipt occurring within 5.25 years post index date, applying the same censoring approach as described above. The regression models adjusted for survivors' sociodemographic characteristics (age at index, sex, region, rurality, health plan type, year of index date) and clinical factors (cancer type, treatment modality) individually (unadjusted analysis), and then combined (multivariable analysis). The proportional hazards assumption of the Cox model was tested using cumulative sums of martingale‐based residuals developed by Lin et al.^14^ and no violation was detected.

## 3 RESULTS

We identified 1609 survivors treated with anthracyclines during childhood and adolescence; 48.4% had attained young adult age (18–28 years) at index date, 47.4% were female, and 13.1% were residing in rural areas (Table 1). The majority of our sample were diagnosed with hematologic cancers (90.3%), followed by bone and soft issue cancers (9.7%). Hematopoietic stem cell transplantation was part of cancer therapies for 5.5% survivors, and 42.4% received chemotherapy alone. The most commonly used anthracycline agent was doxorubicin (93.1%).

**TABLE 1:** Unadjusted and adjusted Cox proportional hazards models estimating time to initial cardiac test.

Characteristics	*n*	Col.%	Unadjusted HR (95% CI)	*p*‐value	Adjusted HR^d^ (95% CI)	*p*‐value
Sociodemographic characteristics						
Age at index date^a^						
0–17 years	831	51.6	Ref		Ref	
18–28 years	778	48.4	0.44 (0.38, 0.52)	<0.001	0.42 (0.35, 0.49)	<0.001
Sex						
Male	846	52.6	Ref		Ref	
Female	763	47.4	1.07 (0.93, 1.25)	0.35	0.99 (0.85, 1.15)	0.85
Geographic region						
Northeast	376	23.4	Ref		Ref	
North central	377	23.4	0.90 (0.73, 1.11)	0.33	0.98 (0.79, 1.21)	0.83
South	558	34.7	0.82 (0.68, 1.00)	0.051	0.85 (0.70, 1.04)	0.12
West	298	18.5	0.67 (0.53, 0.85)	0.001	0.71 (0.56, 0.90)	0.005
Rurality of residence						
Rural	210	13.1	Ref		Ref	
Urban	1399	86.9	1.09 (0.87, 1.37)	0.47	1.10 (0.87, 1.39)	0.43
Health plan type						
High‐deductible plan only	219	13.6	Ref		Ref	
Health maintenance organization only	168	10.4	1.17 (0.87, 1.58)	0.31	1.19 (0.87, 1.62)	0.27
Preferred provider organization only	955	59.4	1.05 (0.83, 1.32)	0.68	1.07 (0.84, 1.35)	0.59
Other or multiple plan types	267	16.6	1.10 (0.83, 1.45)	0.50	1.08 (0.81, 1.44)	0.61
Year at index date^a^						
2009–2010	171	10.6	Ref		Ref	
2011–2012	416	25.9	0.82 (0.63, 1.05)	0.12	0.79 (0.61, 1.02)	0.070
2013–2014	431	26.8	0.91 (0.70, 1.17)	0.44	0.87 (0.67, 1.13)	0.31
2015–2016	337	20.9	0.96 (0.74, 1.25)	0.78	0.84 (0.64, 1.10)	0.21
2017–2018	254	15.8	0.93 (0.68, 1.25)	0.61	0.85 (0.62, 1.16)	0.29
Clinical characteristics						
Site and type of cancer						
Hematologic cancers^b^	1453	90.3	Ref		Ref	
Bone and soft tissue cancers	156	9.7	1.84 (1.47, 2.29)	<0.001	1.64 (1.30, 2.07)	<0.001
Cancer treatment						
Chemotherapy only	682	42.4	Ref		Ref	
Any HSCT	89	5.5	2.00 (1.49, 2.70)	<0.001	2.23 (1.63, 3.03)	<0.001
Any radiation (no HSCT)	475	29.5	1.17 (0.98, 1.40)	0.09	1.42 (1.18, 1.72)	<0.001
Any surgery (no HSCT or radiation)	363	22.6	1.10 (0.90, 1.34)	0.36	1.35 (1.09, 1.67)	0.006
Receipt of any daunorubicin (ref: none)	232	14.4	1.46 (1.21, 1.78)	<0.001	1.24 (0.97, 1.60)	0.09
Receipt of any doxorubicin (ref: none)	1498	93.1	0.68 (0.53, 0.89)	0.005	0.80 (0.56, 1.14)	0.22
Receipt of other types of anthracycline^c^ (ref: none)	32	2.0	0.93 (0.55, 1.58)	0.78	1.00 (0.56, 1.80)	1.00

Abbreviations: CI, confidence interval; HSCT, hematopoietic stem cell transplantation; HR, hazard ratio; Ref, reference.

*Note*: *N* = 1609.

^a^ The index date was defined as 6 months after the end‐of‐therapy date.

^b^ Hematologic cancer includes leukemia and lymphoma. Using insurance claims data, our analysis relied on the International Classification of Diseases (ICD) diagnosis codes, which make it difficult to distinguish subtypes of hematological cancer.

^c^ Other types of anthracyclines include mitoxantrone, idarubicin, epirubicin, and alemtuzumab.

^d^ A multivariable Cox model was estimated that controlled for all covariates listed in this table.

**FIGURE 1:**
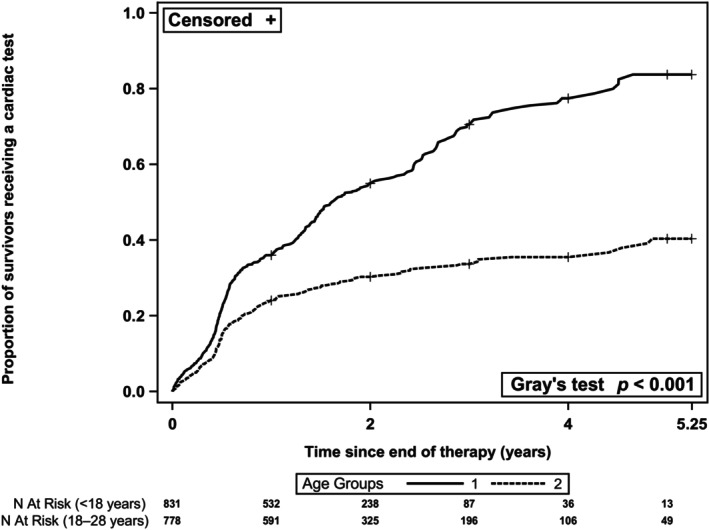
Cumulative incidence curve of time to first cardiac test receipt after index date, by age group. The cumulative incidence of receiving the initial cardiac test (95% confidence interval) at 5.25 years post index date was 83.7% (76.6–88.8%) and 40.3% (34.2–46.4%) for children (<18 years) and young adults (18–28 years), respectively (Gray's test *p* < 0.001).

Overall, the cumulative incidence of receiving an initial cardiac test by 5.25 years post index date was 62.3% (95% confidence interval [CI]: 57.5% to 66.7%), with median time to the initial test receipt being 2.7 years (95% CI: 2.5 to 3.1 years). Among cardiac test recipients (*n* = 694 survivors), the vast majority underwent an echocardiogram (*n* = 691 survivors). When stratified by age at index, the cumulative incidence of receiving an initial cardiac test within 5.25 years post index date remained lower in young adults than children ≤17 years (40.3% [95% CI: 34.2% to 46.4%] vs. 83.7% [95% CI: 76.6% to 88.8%]; Gray's test *p* < 0.001; Figure 1). The cumulative incidence of receiving an initial cardiac test within 5.25 years post index date did not differ significantly by other sociodemographic characteristics, except for geographic region (Figure S2).

In the multivariable regression analysis, young adults were less likely to receive cardiac testing than children (hazard ratio [HR] = 0.42; 95% CI: 0.35 to 0.49; Table 1). More likely to receive cardiac testing were survivors who underwent hematopoietic stem cell transplantation versus chemotherapy only (HR = 2.23; 95% CI: 1.63 to 3.03), and survivors with bone and soft tissue cancers versus hematologic cancers (HR = 1.64; 95% CI: 1.30 to 2.07). In addition, survivors living in the Western region were less likely than those in the Northeastern region to receive a cardiac test (HR = 0.71; 95% CI: 0.56 to 0.90).

## 4 DISCUSSION

This nationwide, claims data‐based study of a privately‐insured cohort showed that nearly 40% of anthracycline‐treated survivors of childhood and adolescent cancer at risk of cardiac dysfunction had not received any cardiac test within 5.25 years following index date, despite national guidelines during the study period detailing the importance of timely screening for early detection of cardiac dysfunction.^15^, ^16^ Our claims data provide precise measurement of realized cardiac testing and new evidence of age differences, with young adult survivors less likely than children to receive cardiac tests despite having insurance.

There are potential explanations for the low uptake of guideline‐recommended cardiac testing. First, the transition into survivorship often involves primary care providers, who may not feel well‐equipped to provide survivorship care or lack familiarity with the guidelines or survivors' therapy risk.^17^, ^18^ Inadequate care coordination between primary care providers and oncology specialists may exacerbate care fragmentation. Second, existing guidelines may be too complex to interpret and lack effective dissemination among providers who are not survivorship focused. Third, survivor‐level challenges—including limited awareness of future health complications, limited knowledge and self‐efficacy to pursue recommended testing and therapies, and logistical barriers including work limitations and travel time—may contribute to low uptake. The psychosocial effects of cancer—such as anxiety, depression, and financial hardship—may further deter survivorship care visits, particularly among young adults. Additionally, young adults face competing responsibilities (e.g., childcare, work schedule) that may limit attention to survivorship care. More research is needed to understand the multi‐level causes of low uptake of cardiac testing, including challenges during young adults' transition from pediatric to adult care.

While our approach delineates feasibility of an algorithm for assessing specific screening service utilization in survivors of a childhood or adolescent cancer,^19^ several study limitations are notable. First, the MarketScan® database contains a convenience sample derived largely from medium‐to‐large employers^8^, ^9^; our findings may not generalize to other childhood and adolescent cancer‐survivor populations in the United States. In particular, our sample could not capture those with public insurance such as Medicaid, the uninsured, and small employers, potentially underrepresenting survivors with lower socioeconomic status who may face additional barriers to accessing cardiac testing.^20^, ^21^, ^22^ Second, these claims data do not provide the cumulative dose of anthracycline or capture tests self‐paid or paid by public insurance. Similarly, our data lacked information on race and ethnicity.

Third, our claims‐based analysis relied on ICD codes that were unable to clearly distinguish hematologic cancer subtypes. Fourth, to minimize sample attrition due to discontinued insurance enrollment over time, we did not assess long‐term use of cardiac testing, an area meriting future investigation. In addition, we lacked information on whether survivors' discontinued enrollment was due to death. Since our model does not account for death as a competing risk, we might have overestimated test receipt rate.^23^ However, sensitivity analysis using a subset of survivors with 5‐year continuous enrollment showed results similar to our main analyses (results available upon request). Finally, as adult guidelines from the American Society of Clinical Oncology are discordant with the COG guidelines,^24^ the recommended time to initiation of young adult cardiac testing remains ambiguous in the absence of symptoms or abnormal echocardiogram.

Our findings underscore the importance of future interventions to improve utilization of guideline‐recommend cardiac tests among survivors of childhood and adolescent cancers. We demonstrate the feasibility of using insurance claims data to identify gaps in real‐world cancer survivorship care. This approach informs future work to assess and implement guideline‐recommended screening for late effects associated with therapies other than anthracycline.

## AUTHOR CONTRIBUTIONS

**Xu Ji:** Conceptualization (equal); data curation (lead); funding acquisition (equal); investigation (equal); methodology (equal); project administration (equal); resources (lead); software (equal); validation (equal); visualization (supporting); writing – original draft (lead); writing – review and editing (equal). **Xin Hu:** Conceptualization (equal); formal analysis (lead); investigation (equal); methodology (equal); software (equal); validation (equal); visualization (lead); writing – review and editing (equal). **Joseph Lipscomb:** Conceptualization (equal); funding acquisition (equal); investigation (equal); methodology (equal); validation (equal); visualization (supporting); writing – review and editing (equal). **Eric J. Chow:** Conceptualization (equal); investigation (equal); methodology (equal); validation (equal); visualization (supporting); writing – review and editing (equal). **Ann Mertens:** Conceptualization (equal); funding acquisition (equal); investigation (equal); methodology (equal); supervision (equal); validation (equal); visualization (supporting); writing – review and editing (equal). **Sharon Castellino:** Conceptualization (equal); funding acquisition (equal); investigation (equal); methodology (equal); project administration (equal); supervision (equal); validation (equal); visualization (supporting); writing – review and editing (equal).

## FUNDING INFORMATION

This work was supported in part by a Pediatric Research Alliance Pilot Grant from the Pediatric Research Alliance and Children's Healthcare of Atlanta (Ji, Mertens, Castellino), and by Award SIP 20–004 from the Centers for Disease Control and Prevention (Ji, Lipscomb, Mertens, Castellino). The content is solely the responsibility of the authors and does not necessarily represent the official views of the Centers for Disease Control and Prevention, the Pediatric Research Alliance, or Children's Healthcare of Atlanta.

## CONFLICT OF INTEREST STATEMENT

Xu Ji, Sharon M. Castellino, Ann C. Mertens reported grants from the National Cancer Institute, National Institute on Minority Health and Health Disparities, Centers for Disease Control and Prevention, Emory University, Rally Foundation for Childhood Cancer Research, and The Leukemia & Lymphoma Society outside the submitted work. Xin Hu received a predoctoral fellowship from the PhRMA Foundation outside the submitted work. The authors have no other conflicts of interest to disclose.

## ETHICS STATEMENT

This study was deemed exempt by the Emory University Institutional Review Board.

## Supporting information


Data S1.

## Data Availability

Data for this analysis was made available to the authors through a third‐party license from Merative, a commercial data provider in the United States. As such, the authors cannot make these data publicly available due to data use agreement. Other researchers can access the data by purchasing a license through Merative. The inclusion criteria specified in the Methods section would allow other researchers to identify the same cohort of patients used for this analysis. Interested individuals may visit https://marketscan.truvenhealth.com/marketscanportal/ for more information on accessing Merative MarketScan databases.
