# Supplementary material for: Utilization of cardiac tests in anthracycline‐treated cancer survivors differs between young adults and children: A claims‐based analysis
Source: Cancer Med. 2023 Dec 9;12(24):22056–61. doi: 10.1002/cam4.6801 (PMC10757126; doi:10.1002/cam4.6801)
Supplement: Supplementary file 1 — Data S1. [file CAM4-12-22056-s001.docx]

**Supplemental Table 1:** Codes to identify anthracyclines

Panel A: Procedure codes for injectable anthracyclines

| Daunorubicin: | |
| --- | --- |
| J9150 | Injection, daunorubicin, 10 mg as maintained by CMS falls under chemotherapy drugs. |
| J9151 | Injection, daunorubicin citrate, liposomal formulation, 10 mg |
| Doxorubicin: | |
| J9000 | Injection, doxorubicin hydrochloride, 10 mg as maintained by CMS falls under chemotherapy drugs |
| J9001 | Injection, doxorubicin hydrochloride, all lipid formulations, 10 [milligrams]. |
| J9002 | Injection, doxorubicin hydrochloride, liposomal, doxil, 10 mg (Terminated on Tuesday, December 31, 2013) |
| Q2048 | Injection, doxorubicin hydrochloride, liposomal, doxil, 10 mg |
| Q2049 | Injection, doxorubicin hydrochloride, liposomal, imported Lipodox, 10 mg as maintained by CMS |
| Q2050 | Injection, doxorubicin hydrochloride, liposomal, not otherwise specified, 10 mg |
| Mitoxantrone: | |
| J9293 | Injection, mitoxantrone hydrochloride, per 5 mg as maintained by CMS falls under Chemotherapy Drugs |
| Idarubicin: | |
| J9211 | Injection, idarubicin hydrochloride, 5 mg. |
| Epirubicin: | |
| J9178 | Injection, epirubicin hcl, 2 mg |
| Other codes used in previous studies to pull claims with anthracycline: | |
| J9010 | Injection, alemtuzumab, 10 mg |

Panel B: Oral anthracycline medications

| Generic Name | Brand Name |
| --- | --- |
| Daunorubicin | Daunorubicin |
| Daunorubicin | DaunoXome |
| Daunorubicin | Vyxeos |
| Doxorubicin | Doxorubicin |
| Doxorubicin | Rubex |
| Epirubicin | Epirubicin/Ellence |
| Idarubicin | Idarubicin |
| Idarubicin | Idamycin |
| Mitoxantrone | Mitoxantrone |

**Supplemental Figure 1:** Sample derivation flowchart


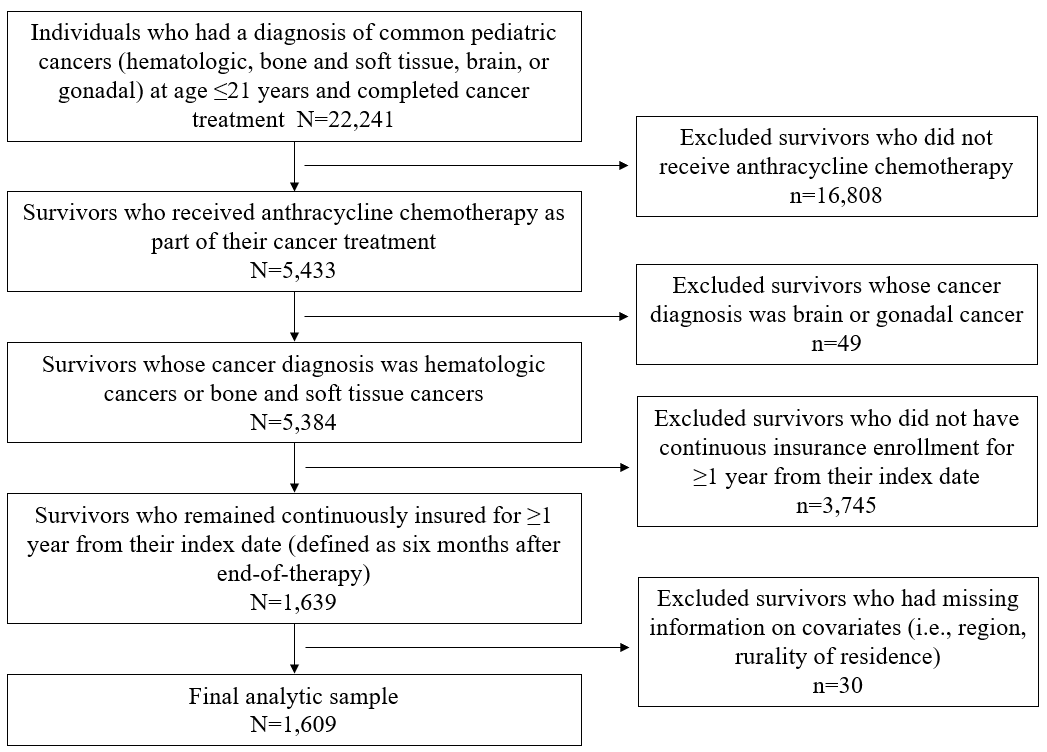


**Supplemental Table 2:** Procedure codes to identify cardiac testing

**Panel A:** Procedure codes for echocardiogram:

| 76825 | Echocardiography, fetal, cardiovascular system, real time with image documentation (2D), with or without M-mode recording; initial |
| --- | --- |
| 76827 | Doppler echocardiography, fetal, pulsed wave and/or continuous wave with spectral display; initial |
| 93303 | Transthoracic echocardiography for congenital cardiac anomalies; complete |
| 93304 | Transthoracic echocardiography for congenital cardiac anomalies; follow-up or limited study |
| 93306 | Echocardiography, transthoracic, real-time with image documentation (2D), includes M-mode recording, when performed |
| 93307 | Echocardiography, transthoracic, real-time with image documentation (2D) with or without M-mode recording; complete |
| 93308 | Echocardiography, transthoracic, real-time with image documentation (2D) with or without M-mode recording; follow-up or limited study |
| 93312 | Echocardiography, transesophageal, real time with image documentation (2D) (with or without M-mode recording); including probe placement, image acquisition, interpretation and report |
| 93314 | Echocardiography, transesophageal, real time with image documentation (2D) (with or without M-mode recording); image acquisition, interpretation and report only |
| 93315 | Transesophageal echocardiography for congenital cardiac anomalies; including probe placement, image acquisition, interpretation and report |
| 93317 | Transesophageal echocardiography for congenital cardiac anomalies; image acquisition, interpretation and report only |
| 93320 | Doppler echocardiography, pulsed wave and/or continuous wave with spectral display (List separately in addition to codes for echocardiographic imaging); complete (Add-on code) |
| 93321 | Doppler echocardiography, pulsed wave and/or continuous wave with spectral display (List separately in addition to codes for echocardiographic imaging); follow-up or limited study (Add-on code) |
| 93325 | Doppler echocardiography color flow velocity mapping (List separately in addition to codes for echocardiography) (Add-on Code) |
| 93350 | Transthoracic Stress Echo, complete |
| 93351 | Transthoracic Stress Echo, complete w/ cont EKG |

**Panel B:** Procedure codes for cardiac magnetic resonance imaging (MRI)

| 75557 | Cardiac magnetic resonance imaging for morphology and function without contrast material. |
| --- | --- |
| 75558 | Cardiac MRI for morphology/function w/o contrast materials; w/flow/velocity quantification; |
| 75560 | Cardiac MRI for morphology/function w/o contrast materials; w/flow/velocity quantification & stress; |
| 75562 | Cardiac MRI for morphology/function w/o contrast materials; followed by contrast materials/further sequences, w/flow/velocity quantification; |
| 75564 | Cardiac MRI for morphology/function w/o contrast materials; followed by contrast materials/further sequences, w/flow/velocity quantification & stress. |
| 75565 | Cardiac MRI for Velocity Flow Mapping |
| 75559 | Cardiac MRI for morphology and function with stress |
| 75561 | Cardiac MRI for morphology and function without stress |
| 75563 | Cardiac MRI for morphology and function with stress |

**Panel C:** Procedure codes for multiple gate acquisition (MUGA) scan:

| 78452 | Myocardial perfusion imaging, tomographic (SPECT); multiple studies, at rest and/or stress and/or redistribution and/or rest reinjection |
| --- | --- |
| 78453 | Myocardial perfusion imaging, planar (including qualitative or quantitative wall motion, ejection fraction by first pass or gated technique, additional quantification, when performed); single study, at rest or stress (exercise or pharmacologic) |
| 78454 | Myocardial perfusion imaging, planar (including qualitative or quantitative wall motion, ejection fraction by first pass or gated technique, additional quantification, when performed); multiple studies, at rest and/or stress (exercise or pharmacologic) and/or redistribution and/or rest reinjection |
| 78472 | Cardiac blood pool imaging, gated equilibrium; planar, single study at rest or stress (exercise and/or pharmacologic), wall motion study plus ejection fraction, with or without additional quantitative processing |
| 78473 | Cardiac blood pool imaging, gated equilibrium; planar. multiple studies, wall motion study plus ejection fraction, at rest and stress (exercise and/or pharmacologic), with or without additional quantification |
| 78494 | Cardiac blood pool imaging, gated equilibrium, spect, at rest, wall motion study plus ejection fraction, with or without quantitative processing |
| 78414 | Non-Imaging Heart Function |
| 78433 | Myocardial imaging, positron emission tomography (PET), combined perfusion with metabolic evaluation study (including ventricular wall motion[s] and/or ejection fraction[s], when performed), dual radiotracer (eg, myocardial viability); with concurrently acquired computed tomography transmission scan |
| 78451 | Myocardial perfusion imaging, tomographic (spect) (including attenuation correction, qualitative or quantitative wall motion, ejection fraction by first pass or gated technique, additional quantification, when performed); single study, at rest or stress (exercise or pharmacologic) |

**Supplemental Figure 2:** Cumulative incidence curves of time to first cardiac test receipt since index date, by other key sociodemographic characteristics

A. By Sex B. By Region


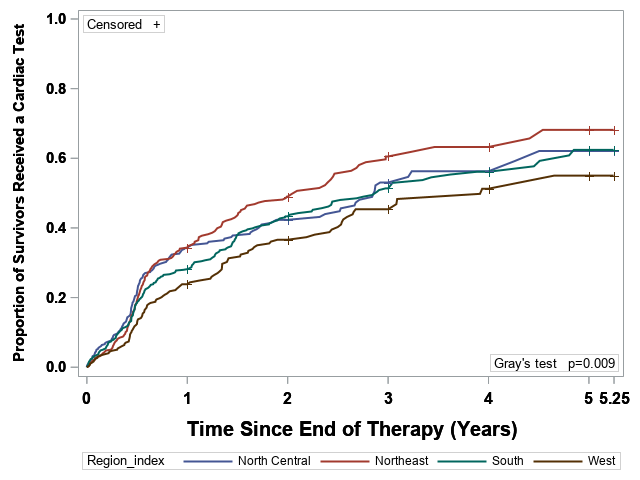


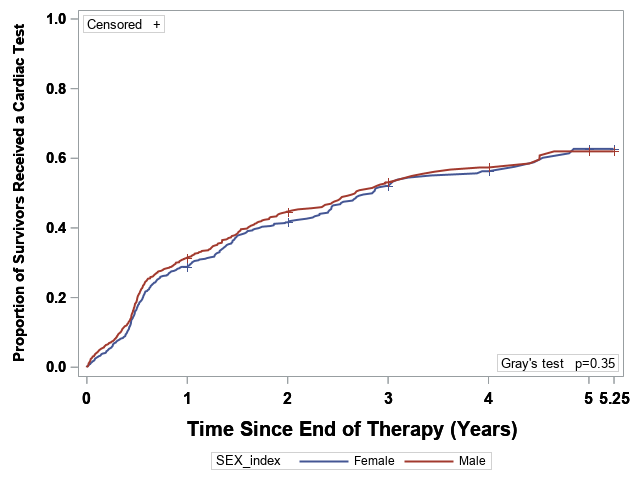


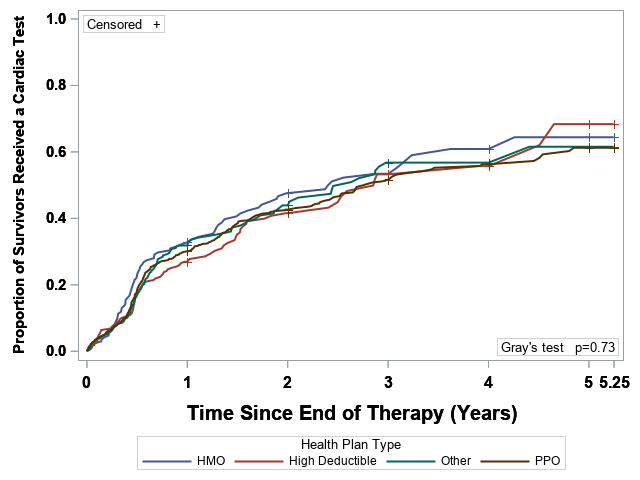

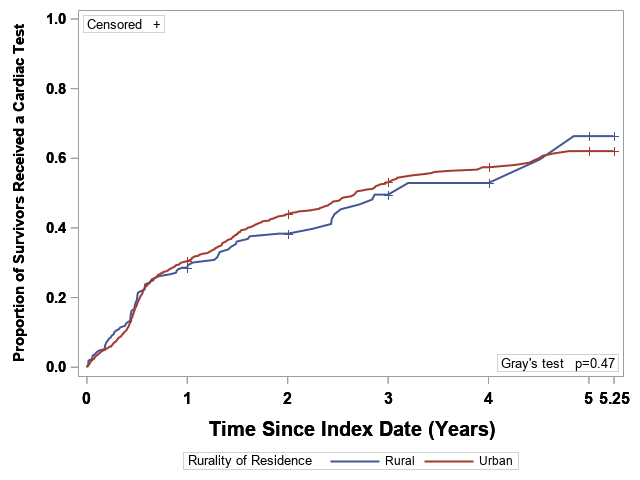
C. By Rurality of Residence D. By Health Plan Type
